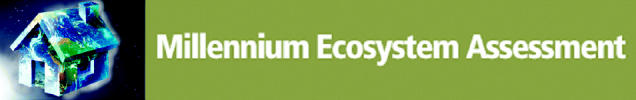# EHPnet: Millennium Ecosystem Assessment

**Published:** 2005-09

**Authors:** Erin E. Dooley

The Millennium Ecosystem Assessment (MA) is the largest assessment to date of the health of the world’s ecosystems. Launched in 2001 by United Nations (UN) secretary-general Kofi Annan and authorized by governments through four international conventions, the MA is intended as a tool to inform decision makers and the public. The documents flowing forth from this work, which was completed in March 2005, have been prepared by 1,360 experts from 95 countries, with an 80-person independent board of review editors. The documents draw on information gathered from the scientific literature, existing data sets, and scientific models, and incorporate knowledge gleaned from the private sector, workers in the field, indigenous peoples, and local communities. Information about the MA, as well as the documents it has released, are available online at **http://www.millenniumassessment.org/**.

The findings of the MA are grim. Over the past 50 years, humans have changed ecosystems faster and more extensively than during any other comparable time period in human history. These rapid changes have grown out of increasing demands for natural goods and services, such as food, fresh water, timber, fiber, and fuel. The MA also finds that ecosystem changes have brought about substantial gains in human well-being and in economic development, but that these gains have come at the cost of degrading ecosystem services and increasing poverty for some groups of people. The report predicts that ecosystem change could accelerate during the next 50 years and contribute to nonachievement of the UN Millennium Development Goals.

Yet, there is some hope that this situation can still be reversed, and the report sets forth options for improving ecosystems by 2050. These fall under three scenarios: “Global Orchestration,” “Adapting Mosaic,” and “TechnoGarden.” The Global Orchestration scenario reflects a globally connected society focused on international trade and economic liberalization that also takes strong steps to reduce problems such as poverty and inequality and to invest in public infrastructure and education. The Adapting Mosaic scenario focuses on local-scale activities, and investments in human and social capital emphasize education to bring about a better understanding of the nature of ecosystems. At the core of the TechnoGarden scenario is the use of technology and highly managed, often engineered ecosystems to deliver ecosystem services. A fourth scenario, “Order from Strength,” emphasizes heightened security and a fragmented society, to the detriment of the environment.

The MA homepage provides the latest news related to the project, while links along the right side of the page access the numerous partners in the MA. These partners include the UN Development Programme, the UN Environment Programme, the World Bank, multiple universities, and others.

The Reports section of the site provides links to the major documents produced by the MA. Each report can be downloaded for free in English and several other languages; there is also information on how to order printed copies. The Resources section assembles slide presentations, figures, tables, maps, posters, logos, and brochures that can be used by the media. All are available to download for free.

The About the MA section of the website provides a thorough history of how the work came about, how it was funded, how it was undertaken, and how it may continue in the future. This section also includes a page devoted to the many subregional assessments that are being carried out in conjunction with the MA. Links to each provide details of the areas covered by the assessments, the institutions carrying out the assessments, the features of the ecosystem being assessed, key features of the assessments, and the time frame and budget for the work.

## Figures and Tables

**Figure:**